# Interval Laparoscopic Cholecystectomy in the Management of Acute Biliary Pancreatitis

**DOI:** 10.1155/2000/40290

**Published:** 2000-01

**Authors:** M. D. Pinhas Schachter, M. D. Timor Peleg, M. D. Oded Cohen

**Affiliations:** 1 Department of Surgery A Wolfson Medical Center Holon and Sackler School of Medicine Tel-Aviv University Haifa Israel; 2 Department of Surgery A Carmel Medical Center Haifa Israel

## Abstract

The timing of laparoscopic cholecystectomy following an attack of acute biliary pancreatitis is controversial. The traditional approach of interval cholecystectomy has been challenged recently. The present study was designed to evaluate the benefits of interval laparoscopic cholecystectomy for patients with mild acute pancreatitis (Ranson less than 3). Nineteen patients with mild pancreatitis underwent ultrasonographic evaluation to confirm the biliary etiology. ERCP was performed in all patients on the first available endoscopy list, and endoscopic sphincterotomy was performed in two patients with calculi or dilated common bile duct on ultrasonographic examination. Medical treatment was administered and laparoscopic cholecystectomy was scheduled after 8–12 weeks to allow the inflammatory process to settle. There were no recurrent attacks of pancreatitis during this period. The degree of difficulty of the laparoscopic procedure was assessed by the presence of adhesions to the gallbladder area, difficulty of dissection in the Calot's triangle, intraoperative bleeding and the need for a drain. Six patients (31.5%) had severe adhesions, difficult dissection of the cystic duct and artery, bleeding and prolonged operating time. In two of these patients (10.5%) the procedure was converted to open cholecystectomy. In conclusion, our results suggest that postponing laparoscopic cholecystectomy in acute pancreatitis patients is not advantageous surgically and does not justify the risk of further morbidity caused by the gallbladder disease.


HPB Surgery, 2000, Vol. 11, pp. 319-323
Reprints available directly from the publisher
Photocopying permitted by license only

© 2000 OPA (Overseas Publishers Association) N.V.
Published by license under
the Harwood Academic Publishers imprint,
part of The Gordon and Breach Publishing Group.
Printed in Malaysia.

### Interval Laparoscopic Cholecystectomy in the Management of Acute Biliary Pancreatitis

M. D. PINHAS SCHACHTER[a,*], M. D. TIMOR PELEG[b] and M. D. ODED COHEN[b]

[a] *Department of Surgery A, Wolfson Medical Center, Holon and Sackler School of Medicine, Tel-Aviv University;*
[b] *Department of Surgery A, Carmel Medical Center, Haifa*

*(Received 1 August 1998; In final form 10 December 1999)*

**The timing of laparoscopic cholecystectomy following an attack of acute biliary pancreatitis is controversial. The traditional approach of interval cholecystectomy has been challenged recently. The present study was designed to evaluate the benefits of interval laparoscopic cholecystectomy for patients with mild acute pancreatitis (Ranson less than 3). Nineteen patients with mild pancreatitis underwent ultrasonographic evaluation to confirm the biliary etiology. ERCP was performed in all patients on the first available endoscopy list, and endoscopic sphincterotomy was performed in two patients with calculi or dilated common bile duct on ultrasonographic examination. Medical treatment was administered and laparoscopic cholecystectomy was scheduled after 8-12 weeks to allow the inflammatory process to settle. There were no recurrent attacks of pancreatitis during this period. The degree of difficulty of the laparoscopic procedure was assessed by the presence of adhesions to the gallbladder area, difficulty of dissection in the Calot's triangle, intraoperative bleeding and the need for a drain. Six patients (31.5%) had severe adhesions, difficult dissection of the cystic duct and artery, bleeding and prolonged operating time. In two of these patients (10.5%) the procedure was converted to open cholecystectomy. In conclusion, our results suggest that postponing laparoscopic cholecystectomy in acute pancreatitis patients is not advantageous surgically and does not justify the risk of further morbidity caused by the gallbladder disease.**

*Keywords:* Laparoscopic cholecystectomy, biliary pancreatitis

### INTRODUCTION

Gallstones and biliary sludge are the most common etiology of acute pancreatitis [1-3]. Cholecystectomy is mandatory to avoid recurrence, whereas the timing of surgery is still controversial [4]. Laparoscopic cholecystectomy is now the standard procedure in the management of gallbladder stones, but experience in instances of acute pancreatitis is limited [5-7]. Moreover, in a multicenter study, most surgeons regarded acute pancreatitis as a contraindication for laparoscopic cholecystectomy [8], while others advocate early surgery, performed shortly after resolution of acute pancreatitis, pointing out the increased technical difficulty of the laparoscopic method [3]. The wide acceptance of endoscopic retrograde cholangiopancreatography (ERCP) and endoscopic sphincterotomy for urgent decompression of the biliary system [9-13], enables the surgeon to plan an elective (interval)

*Corresponding author. Tel.: 972-3-5028607, Fax: 972-9-9586544.

laparoscopic cholecystectomy for the residual gallstones.

With this background we have evaluated our experience with acute pancreatitis patients predicted to have mild disease, treated conservatively initially and having interval laparoscopic cholecystectomy performed within 8–12 weeks from the attack.

### PATIENTS AND METHODS

All patients presenting to our department between January 1995 and June 1997 with acute biliary pancreatitis were evaluated for participation in the study. Overall, about 40–50 patients with acute pancreatitis are hospitalized yearly in the department. Only patients that signed an informed consent were included in the study.

Acute pancreatitis was defined as acute abdominal pain with elevated serum and/or urine levels of amylase (serum levels > 700 IU/l, normal range 70–220 IU/l, urine levels >1500 IU/l, normal < 1000 IU/l). Abdominal ultrasonography was performed on admission to determine the biliary etiology and confirm the diagnosis. Patients were stratified according to the severity of the disease by Ranson's criteria and included in the study only if there was evidence of gallbladder disease, no other etiologic cause of pancreatitis, with Ranson's score of 3 or less, (mild pancreatitis) and they did not have previous abdominal surgery (except appendectomy). Treatment was according to a standard protocol with no oral intake, nasogastric drainage, intravenous fluids and oxygen mask if required. No drugs were prescribed except for non-narcotic analgesia.

Endoscopic retrograde cholangiopancreatography (ERCP) was performed in all patients with acute pancreatitis on the first available endoscopy list. Endoscopic sphincterotomy was performed when the common bile duct was dilated with calculi or sludge demonstrated on ultrasonographic examination. If the initial ERCP failed it was repeated on an elective basis prior to surgery. Clinical improvement was defined as a reduction of abdominal pain and tenderness, normalization of laboratory values and neutral fluid balance. Oral diet was reintroduced gradually, the patients were discharged for an elective laparoscopic cholecystectomy 8–12 weeks later.

Surgery was performed by two experienced laparoscopists (P.S. and O.C.) using a three-cannula technique. The cystic duct and cystic artery were separately ligated with metal clips and the gallbladder was removed through the supraumbilical incision, which was dilated if necessary. The degree of difficulty of the procedure was determined by the presence of adhesions to the gallbladder area, difficulty of dissection in the Calot's triangle, intraoperative bleeding, and the need for a drain. Following surgery, patients were managed with mobilization and reintroduction of diet as soon as tolerated.

### RESULTS

During the study period, 19 patients (4 males and 15 females, age range 20–79 years, mean 59 years) with mild acute biliary pancreatitis qualified for the study.

Abdominal ultrasonography revealed a thickened gallbladder wall (more than 3 mm) in 14 patients. A dilated common duct (over 6 mm) and suspected choledocholithiasis were found in two patients. These two patients underwent ERCP with endoscopic sphincterotomy and clearance of the stones and sludge from the common duct.

ERCP was successful in all patients, initially in 14 patients with acute pancreatitis and was repeated prior to surgery in the remaining 5 patients.

There were no complications related to the ERCP.

Laparoscopic surgery was performed after an interval of 8–12 weeks to allow the acute

TABLE I Operative difficulty for interval laparoscopic cholecystectomy

| | Acute pancreatitis (19 patients) |
|---|---|
| Gallbladder adhesions | 4 (21%) |
| Difficulty of dissection of Calot's triangle | 3 (15.8%) |
| Bleeding | 1 (5.3%) |
| Need for drain | 1 (5.3%) |
| Conversion | 2 (10.5%) |

inflammation to settle. Table I presents the operative difficulty for laparoscopic cholecystectomy. Two patients underwent conversion to open surgery due to severe adhesions that prevented a satisfactory exposure of the anatomy in the Calot's triangle (one patient), and bleeding during the dissection of the cystic duct and artery (one patient). Another four patients of this group had marked adhesions, difficult dissections in the Calot's triangle and prolonged laparoscopic cholecystectomies. In one instance a drain was placed in the area of the cystic artery remnant due to unsatisfactory identification.

The postoperative course was uneventful with a hospitalization stay of 2–5 days.

### DISCUSSION

Acute biliary pancreatitis, is among the most commonly encountered complications of gallbladder stones and sludge. The majority of patients suffer mild attacks that respond promptly to medical treatment. Cholecystectomy is indicated to prevent recurrence. Laparoscopic cholecystectomy is now the procedure of choice, and unless absolute contraindications exist (uncorrectable coagulopathy or concurrent diseases requiring laparotomy) all cholecystectomies are performed laparoscopically [14]. Biliary pancreatitis in the acute phase, has been considered a relative contraindication to the laparoscopic approach as it increases the likelihood of conversion, the operative difficulty and operating time [3, 4, 15, 16]. Moreover, ERCP with ES has become an important and very successful adjunctive in the management of acute biliary pancreatitis, offering the surgeon the possibility to postpone the intervention for several weeks and allow the acute inflammation to settle. Therefore, even in spite of the fact that approximately one-quarter of patients have symptomatic recurrence within 6 weeks if not operated, and the rate increases with time [17–19], the old concept of interval laparoscopic cholecystectomy has regained interest. Indeed, during the 8–12 weeks of delay there were no recurrent attacks in the patients of our group, since adequate drainage was demonstrated on ERCP, or created by the endoscopic sphincterotomy. However, the results of our study, although based on small but carefully selected patient series, suggest that delayed (interval) laparoscopic cholecystectomy does not offer any benefit compared to reports of early interventions [3, 4]. The conversion rate was relatively high – 10% for acute pancreatitis group, compared to our conversion rate for elective laparoscopic cholecystectomies – 2.6% (8 from 303 laparoscopic cholecystectomies performed during the same period of the study). Moreover, dense, vascular adhesions to the gallbladder, a thick-walled gallbladder and difficult dissection in the Calot's triangle causing bleeding and prolonging operating time significantly were recorded in 6 patients of the acute pancreatitis group (including the conversion cases). We do not share the experience described of a dilated cystic duct that did not fit the standard ligating clip and required externally tied Roeder slipknot ligation [3]. The dilated cystic duct was encountered in the majority of patients operated early after the acute inflammation, therefore postponing surgery for 8–12 weeks, and ERCP with or without ES to ensure biliary drainage might explain the non-dilated cystic ducts in our series. The role of intra-operative cholangiography is controversial. In the presence of non-dilated bile ducts on ultrasonography, a normal ERCP or ES and with the knowledge that ERCP

is feasible, we do not feel intra-operative cholangiography is essential.

In conclusion, our results suggest that the benefits obtained by interval laparoscopic cholecystectomy after acute pancreatitis are insufficient to justify the risk of a further episode of acute pancreatitis, although early ERCP and ES are associated with a high success rate and low morbidity. In accordance with our results we recommend surgery during the same hospitalization, as soon as the signs and symptoms of acute pancreatitis have settled.

### *References*

[1] Steinberg, W. and Tenner, S. (1994). Acute Pancreatitis. *N. Engl. J. Med.*, **330**(17), 1198–1210.
[2] Corfield, A. P., Cooper, M. J. and Williamson, R. C. N. (1985). Acute pancreatitis: a lethal disease of increasing incidence. *Gut.*, **26**, 724–729.
[3] Tate, J. J. T., Lau, W. Y. and Li, A. K. C. (1994). Laparoscopic cholecystectomy for biliary pancreatitis. *Br. J. Surg.*, **81**, 720–722.
[4] Liu, C. L., Lo, C. M. and Fan, S. T. (1997). Acute biliary pancreatitis: Diagnosis and Management. *World J. Surg.*, **21**, 149–154.
[5] Fletcher, D. R., Jones, R. M., O'Riordan, B. and Hardy, K. J. (1992). Laparoscopic cholecystectomy for complicated gallstone disease. *Surg. Endosc.*, **6**, 179–182.
[6] Cooperman, A. M., Siegel, J., Neff, R., Reddy, S. and Hammerman, H. (1991). Gallstone pancreatitis: combined endoscopic and laparoscopic approaches. *J. Laparoendoscopic Surg.*, **1**, 115–117.
[7] Rhodes, M., Armstrong, C. P., Longstaff, A. and Cawthorn, S. (1993). Laparoscopic cholecystectomy with endoscopic retrograde cholangiopancreatography for acute gallstone pancreatitis. *Br. J. Surg.*, **80**, 247–249.
[8] Cushieri, A., Dubois, F., Mouiel, J., Mouret, P., Becker, H., Buess, G., Trede, M. and Troidl, H. (1991). The European experience with laparoscopic cholecystectomy. *Am. J. Surg.*, **161**, 385–388.
[9] Folsch, U. R., Nitsche, R., Ludtke, R., Hilgers, R. A. and Creutzfeldt, W. (1997). Early ERCP and papillotomy compared with conservative treatment for acute biliary pancreatitis. *N. Engl. J. Med.*, **336**(4), 237–242.
[10] Lai, E. C. S., Lo, C. M., Choi, T. K., Cheng, W. K., Fan, S. T. and Wong, J. (1989). Urgent biliary decompression after endoscopic retrograde cholangiopancreatography. *Am. J. Surg.*, **157**, 121–125.
[11] Neoptolemos, J. P., London, N. J., James, D., Carr-Locke, D. L., Bailey, I. A. and Fossard, D. P. (1988). Controlled trial of urgent endoscopic retrograde cholangiopancreatography and endoscopic sphincterotomy *versus* conservative treatment for acute pancreatitis due to gallstones. *Lancet.*, **8618**, 979–983.
[12] Fan, S. T., Lai, E. C. S., Mok, F. P. T., Lo, C. M., Zheng, S. S. and Wong, J. (1993). Early treatment of acute biliary pancreatitis by endoscopic papillotomy. *N. Engl. J. Med.*, **328**, 228–232.
[13] Neoptolemos, J. P., Carr-Locke, D. L., London, N. J., Bailey, I. A. and Fossard, D. P. (1988). ERCP findings and the role of endoscopic sphincterotomy in acute gallstone pancreatitis. *Br. J. Surg.*, **75**, 954–960.
[14] Soper, N. J., Brunt, I. M. and Kerbl, K. (1994). Laparoscopic general surgery. *N. Engl. J. Med.*, **330**(6), 409–419.
[15] Soper, N. J. (1991). Laparoscopic cholecystectomy. *Curr. Probl. Surg.*, **28**, 585–655.
[16] Tang, E., Stain, S. C., Tang, G., Froes, E. and Berne, T. V. (1995). Timing of laparoscopic surgery in gallstone pancreatitis. *Arch. Surg.*, **130**, 496–499.
[17] Semel, L., Schreiber, D. and Fromm, D. (1983). Gallstone pancreatitis: support for a flexible approach. *Arch. Surg.*, **118**, 901–904.
[18] Frei, G. J., Frei, V. T., Thirlby, R. C. and McClelland, R. N. (1986). Biliary pancreatitis: clinical presentation and surgical management. *Am. J. Surg.*, **151**, 170–174.
[19] Pellegrini, C. A. (1993). Surgery for gallstone pancreatitis. *Am. J. Surg.*, **165**, 515–518.

### INVITED COMMENTARY ON

Schachter P, Peleg T, Cohen, O: "Interval laparoscopic cholecystectomy in the management of acute biliary pancreatitis"

Dr. Ronnie Tung-Ping Poon
and Prof. Sheung-Tat Fan
Department of Surgery
The University of Hong Kong Medical Centre
Queen Mary Hospital
Hong Kong
China

### COMMENTARY

The timing of cholecystectomy after acute gallstone pancreatitis is an important but unresolved issue. A few studies in the open cholecystectomy era, including one prospective randomized trial, have shown that early cholecystectomy within the same admission after resolution of pancreatitis was preferable as it avoided the risk of recurrent pancreatitis, while the operative morbidity and mortality were comparable to interval cholecystectomy [1–4]. However, the management of gallstone pancreatitis has undergone major changes over the past decade. First, early endoscopic retrograde cholangiopancreatography (ERCP) and papillotomy have become widely accepted in the management of gallstone pancreatitis. Second, laparoscopic cholecystectomy has emerged as the standard operation for gallstone disease, even for patients presenting with pancreatitis. Hence there is a need to re-evaluate the optimal timing for cholecystectomy in the laparoscopic era.

The authors of this study attempted to address this issue by evaluating their results of interval laparoscopic cholecystectomy for gallstone pancreatitis. A few previous studies of early laparoscopic cholecystectomy after pancreatitis have reported increased technical difficulties due to associated inflammatory changes, and hence a high conversion rate of 12–24% [5–8]. The authors of the current study have found that interval cholecystectomy 8–12 weeks after resolution of pancreatitis was also associated with significant technical difficulties due to adhesions, and the conversion rate was 10% among 19 patients. This conversion rate was relatively high compared with 2.6% in their elective laparoscopic cholecystectomies, presumably for uncomplicated gallstone disease, and the authors concluded that interval cholecystectomy was not advantageous. The validity of the data was somewhat weakened by the small sample size. Their conclusion that early laparoscopic cholecystectomy in the same admission should be recommended could not be fully justified without any results of early operation in their study, although they did draw a comparison with previous reports of early laparoscopic cholecystectomy after pancreatitis. Nevertheless, this study is worthy of inclusion in the literature, as little data on the results of interval laparoscopic cholecystectomy for gallstone pancreatitis were available.

The risk of recurrent pancreatitis or other complications of gallstone disease is the main argument against interval cholecystectomy. The authors of this study have quoted from the literature a 25% risk of symptomatic currence within six weeks, but the figure was derived from studies performed when early ERCP has not yet become a practice in the management of gallstone pancreatitis. The risk of recurrent pancreatitis would presumably be reduced by early endoscopic removal of common bile stones. In fact, the authors found no recurrent attacks of pancreatitis in their patients. A previous study of 48 patients with gallstone pancreatitis who had undergone early ERCP followed by elective laparoscopic cholecystectomy in our institution also demonstrated no interim attacks of pancreatitis [9]. These results suggested that the risk of recurrent pancreatitis was minimal with early ERCP and papillotomy.

The authors of this study have to be commended for their effort to resolve the question of the optimal timing for laparoscopic cholecystectomy after gallstone pancreatitis, but the controversy could not be settled without a prospective randomized study. A randomized trial of early *versus* interval laparoscopic cholecystectomy is under way in our department, and it is hoped that a more definite answer could be provided after the conclusion of the trial.

### *References*

[1] Ranson, J. C. (1979). Timing of biliary surgery in acute pancreatitis. *Ann. Surg.*, **189**, 654–662.

[2] Osborne, D. H., Imrie, C. W. and Carter, D. C. (1981). Biliary surgery in the same admission for gallstone associated acute pancreatitis. *Br. J. Surg.*, **68**, 758–61.

[3] Kelly, T. R. and Wagner, D. S. (1988). Gallstone pancreatitis: a prospective randomized trial of timing of surgery. *Surgery*, **104**, 600–603.

[4] Burch, J. M., Feliciano, D. V., Mattox, K. L. and Jordan, G. L. Jr. (1990). Gallstone pancreatitis. The question of time. *Arch. Surg.*, **125**, 853–860.

[5] Taylor, E. W., Dunham, R. H. and Bloch, J. H. (1994). Laparoscopic management of gallstone pancreatitis. *J. Laparoendosc Surg.*, **4**, 121–125.

[6] Tate, J. J. T. and Arthur, W. Y. (1994). Li. Laparoscopic cholecystectomy for biliary pancreatitis. *Br. J. Surg.*, **81**, 720–722.

[7] Tang, E., Stain, S. C., Tang, G., Froes, E. and Berne, T. V. (1995). Timing of laparoscopic cholecystectomy in gallstone pancreatitis. *Arch. Surg.*, **130**, 496–500.

[8] Bulkin, A. J., Tebyani, N. and Dorazio, R. A. (1997). Gallstone pancreatitis in the era of laparoscopic cholecystectomy. *Am. Surg.*, **63**, 900–903.

[9] Liu, C. L., Lo, C. M. and Fan, S. T. (1997). Acute biliary pancreatitis: diagnosis and management. *World J. Surg.*, **21**, 149–154.

